# Chitosan-Modified Biochar and Unmodified Biochar for Methyl Orange: Adsorption Characteristics and Mechanism Exploration

**DOI:** 10.3390/toxics10090500

**Published:** 2022-08-27

**Authors:** Nguyen Xuan Loc, Phan Thi Thanh Tuyen, Le Chi Mai, Do Thi My Phuong

**Affiliations:** 1 Department of Environmental Sciences, College of the Environment and Natural Resources, Can Tho University, Can Tho 900000, Vietnam; 2 Department of Environmental Engineering, College of the Environment and Natural Resources, Can Tho University, Can Tho 900000, Vietnam

**Keywords:** adsorption, biochar, chitosan, modification, rice husk, methyl orange

## Abstract

In this study, shrimp shell-derived chitosan (CS) and rice husk-derived biochar (RHB) were produced; CS and RHB were then used to synthesize chitosan-modified biochar (CSBC) hydrogel beads. N_2_ adsorption (77K), SEM-EDX and FT-IR techniques were used to evaluate the physicochemical properties of the adsorbents. A batch experiment was conducted to test the methyl orange (MO) adsorption performance of RHB and CSBC. The results showed that the MO adsorption process was strongly pH-dependent. The kinetics were well described by the pseudo-second-order and intra-particle diffusion models, assuming the chemisorption and intraparticle diffusion mechanisms govern the adsorption process. Homogeneous adsorption for MO on the surface of RHB and CSBC was also assumed since the isotherm data showed the best-fit to the Langmuir model. Under the experimental conditions of initial pH 3, dosage 0.2 g, contact time 240 min and temperature 298 K, the maximum adsorption capacity of CSBC and RHB for MO dye adsorption was 38.75 mg.g^−1^ and 31.63 mg.g^−1^, respectively. This result demonstrated that biochar had better performance after modification with chitosan, which provided more functional groups (i.e., −NH_2_ and −OH groups) for enhanced electrostatic interactions and complexation between MO and CSBC. Overall, CSBC is an effective adsorbent for the removal of MO from aqueous solution.

## 1. Introduction

Dye, a complex and soluble organic compound, is commonly used to impart color to fibrous materials. Worldwide, more than 10,000 forms of dyes and an estimated 700,000 tons are produced every year [1,2]. Particularly in the textile industry, roughly 200,000 tons of dyes are annually expelled to effluents during production and application [1]. The high-colored effluents are highly toxic to the environment and humans if they discharge into water bodies without treatment. Most complex synthetic dyes can persist as stable aquatic pollutants, impede the photosynthetic activity of algae, cause depletion of dissolved oxygen in water bodies and thereby pose a threat to aquatic species. They are also categorized as possibly toxic, mutagenic and carcinogenic to humans [3]. Methyl orange (MO) is a common water-soluble and typical azo anionic dye (commonly known as a pH indicator) that is one of the most common dyes used in the textile industry [4,5]. MO contains aromatic and –N = N− groups in its molecules, which are highly toxic, carcinogenic, teratogenic and harmful to the environment and organisms [6]. Thus, MO is identified as a potential organic pollutant that requires proper treatment before its discharge into natural water bodies.

Various physical and chemical biological methods have been employed for the removal of dyes from textile effluents, such as the advanced oxidation process [6], membrane filtration [7], electrochemical oxidation [8], ozonation [9] and coagulation/flocculation-based methods [10]. However, these techniques have their own limitations in terms of complicated design, low removal performance and high operating and maintenance costs. Adsorption is a widely accepted technology due to its high effectiveness, easy operation, inexpensive cost and the possibility of scaling-up to an industrial scale from a laboratory or a pilot scale [11]. Nevertheless, the efficiency of an adsorption process is largely dependent on the adsorbent used in the process [12]. A variety of adsorbents have been developed and used for dye removal, including activated carbon [13], zeolite [14], biochar [15], clay [16], metal–organic framework [17], natural materials [18], agricultural wastes [19] and synthesized products [20]. Biochar-based materials have been widely endorsed as effective and eco-friendly adsorbents for the removal of a range of heavy metals and organic contaminants thanks to their high surface functional groups, high porous structure and large surface area [21,22]. A study by Bussan et al. (2016) used biochar to reduce mercury methylation rates in aquatic sediments [23]. Nevertheless, the heterogeneous nature, high pH and negatively charged surface of biochar make biochar an inconsistent adsorbent [24]. To address this issue, a number of physical, mechanical and chemical modification processes of biochar have been developed in recent years [25]. Many studies have shown that the incorporation of foreign materials (such as zeolite, silica, polymers and metal oxides) into the biochar matrix will enhance functional groups, recalcitrance potential and adsorption efficiencies of the precursor biochar [26,27]. For instance, Fang et al. (2020) used Mg/Ca-modified biochar from peanut shells and achieved a high amount of phosphate (129.79 mg.g^−1^) to be removed from the acid-extract of incinerated sewage sludge ash [28]. In our previous study, a higher adsorption (41.59 mg.g^−1^) of Safranin O was recorded using rice straw biochar modified with iron oxide [29].

Chitosan is a biodegradable and renewable polymer with amazing characteristics of abundantly availability, cost-effectiveness, good biocompatibility and biodegradation, nontoxic nature and a broad range of applications [30]. This polymer is derived from a nitrogenous polysaccharide called chitin, the second most abundant natural polysaccharide, which is extracted from the exoskeleton of invertebrates such as insects or crustaceans. Chitosan is a linear copolymer of β-1,4-linked 2-amino-2-deoxy-β-D-glucose (deacetylated D-glucosamine) and N-acetyl-D-glucosamine units. In general, chitosan possesses two essential functional groups, including (1) free amino (-NH_2_) groups (existing in the D-glucosamine units) and (2) free hydroxyl (-OH) groups (attaching to both the N-acetyl-D-glucosamine units and D-glucosamine units). These groups can take on a positive charge (NH_3_^+^ or H_3_O^+^) which creates a good chelating ligand capable of binding to metal ions [31] or electrostatic attraction to dye anions [32]. Therefore, chitosan has also received much attention as a promising adsorbent to remove metal ions [33,34] or ionic dyes [35,36] from aqueous solution.

There is increasing interest in modifying biochar with foreign materials such as chitosan since this modification benefits from the combined advantages provided by biochar (high porous network and large surface area) and chitosan (high chemical affinity) [37]. Furthermore, chitosan can be utilized as an organic glue to attach pollutants on the surface of biochar and thus, the adsorption capacities of biochar modified by chitosan to various pollutants are generally improved [38]. For example, chitosan-coated onto biochar surfaces showed an adsorption capacity for phloridzin (28.96 mg.g^−1^), nearly double the unmodified biochar derived from apple branches (15.93 mg.g^−1^) [39]. Additionally, the modification of chitosan on biochar surface has been shown to have greater adsorption capacities for Cd(II) and Pb(II) ions than unmodified biochar [40,41].

The adsorption applications of chitosan-modified biochar hydrogel beads to heavy metal ions have been explored in several studies [42,43,44]. For dye ions, chitosan-modified biochar hydrogel beads used for Malachite Green and Rhodamine B removal have also been reported [45]. However, the effectiveness of chitosan-modified biochar hydrogel beads for methyl orange removal from contaminated aqueous media has yet to be explored. Therefore, in this study, biochar was prepared from rice husk, chitosan was derived from shrimp shell and then rice husk biochar (RHB) was modified with chitosan (CS) to form chitosan-modified biochar (CSBC) hydrogel beads. The physicochemical characteristics of the synthesized RHB and CSBC were first evaluated using different techniques and measurements, including SEM, EDX, FTIR techniques, N_2_ adsorption (77 K) and pH_pzc_ measurements. Then, their adsorption capacities toward methyl orange from aqueous solutions were tested using batch experiments, where the pH solution, the mass of the adsorbent, the contact time and the initial MO concentration were varied. Finally, the adsorption mechanism was explored using various isotherm and kinetic models.

## 2. Materials and Methods

### 2.1. Chemicals

All chemicals, including acetic acid (CH_3_COOH), hydrochloric acid (HCl) and sodium hydroxide (NaOH), were provided by Merck (Germany). Methyl orange (MO) was supplied by Sigma-Aldrich.

### 2.2. Chitosan, Biochar and Chitosan-Modified Biochar Production

**Figure 1:**
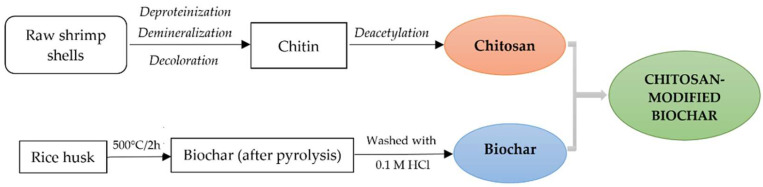
Schematic diagram of preparation process of chitosan-modified biochar.

To synthesize chitosan-modified biochar, shrimp shell-derived chitosan and rice husk-derived biochar were firstly produced, and then biochar was modified with chitosan to form chitosan-modified biochar hydrogel beads following the diagram displayed in Figure 1. For the extraction of chitosan from shrimp shells, raw material *Penaeus monodon* shrimp shell waste was collected from a local seafood company in the Mekong Delta and the procedure of Radwan et al. (2012) was followed [46]. For the pyrolysis of biochar from rice husk, raw rice husk (*OM5451* rice variety) was also collected from a local rice-milling factory in the Mekong Delta and the procedure provided in ref. [29] was followed. The solid collected at the end of this step was identified as rice husk biochar (RHB). Finally, chitosan-modified biochar adsorptive beads were prepared following the protocol summarized by Dewage et al. (2018) [43]. The hydrogel beads collected at the end of this step were identified as chitosan-modified biochar (CSBC).

### 2.3. Characterization of Adsorbents

The micrographs and elemental compositions of the RHB and CSBC adsorbents were evaluated by scanning electron microscopy with energy dispersive X-ray spectroscopy (SEM-EDX Hitachi S4800, Hitachi Ltd., Tokyo, Japan), while their functional groups were analyzed using Fourier transform infrared spectroscopy (FTIR-PerkinElmer Spectrum 10.5.2). The textural characterization of RHB and CSBC was obtained from the nitrogen adsorption–desorption isotherms at 77 K in a Nova Station A (Quantachrome Instruments version 11.0, Miami, FL, USA). The point of zero charge (pH_pzc_) was examined for the pH range of 2.0 to 12.0 using 0.1 M NaCl [25].

### 2.4. Sorption Batch Trials

The batch adsorption experiments were used to evaluate the effect of solution pH (3–10), adsorbent dose (0.1–0.5 g), initial MO concentration (10–200 mg.g^−1^) and adsorption time (1–720 min). The adsorption experiment was performed using #15 mL conical centrifuge tubes, in which a fixed amount of RHB or CSBC was added to a 10 mL MO solution. The mixtures were then agitated in a rotary shaker (HS 250 Basic, IKA Labortechnik) at an agitation speed of 120 rpm and under room conditions (25 ± 2 °C) for a fixed time. After filtering with Whatman No. 6 filter paper, UV-Vis spectroscopy (Shimazdu UV-1900, Kyoto, Japan) was used to analyze the residual MO in the remaining solution.

The amount of MO adsorbed (*q_e_*, mg.g^−1^) was determined according to the following Equation (1):

(1) $$q_{e}=\frac{C_{0}-C_{e}}{m}V,$$

where *C*_0_ (mg.L^−1^) is the initial MO concentration; *C_e_* (mg.L^−1^) is the MO concentration at equilibrium; m (g) is the mass of adsorbent; *V* (mL) is the volume of the solution.

All the experiments in this study were conducted in triplicate (*n* = 3), and data were expressed as the mean ± standard deviation.

#### 2.4.1. Kinetic Modelling

Pseudo-first-order, pseudo-second-order and intraparticle diffusion models were used to analyze the kinetic data of MO on RHB and CSBC. Their equations are shown in Equations (2)–(4):

Pseudo-first-order: *q_t_ = q_e_(1 − exp^−k1t^)*, (2)

where *q_e_* (mg.g^−1^) is the adsorption capacity at equilibrium; *q_t_* (mg.g^−1^) is the adsorption capacity at a given *t* time; *k_1_* (1.min^−1^) is the constant rate.

(3) $$\mathrm{Pseudo}\text{-}\mathrm{second}\text{-}\mathrm{order}:\;\frac{dq_{t}}{dt}=k_{2}{\left(q_{e}-q_{t}\right)}^{2},$$

where *k_2_* (g.mg^−1^.min^−1^) is the constant rate.

Intraparticle diffusion: *q_t_ = k_id_t^1/2^ + C*, (4)

where *C* is the intercept; *k_id_* (mg.g^−1^.min^−1/2^) is the intraparticle diffusion rate constant.

#### 2.4.2. Isotherm Modelling

The equilibrium adsorption data were simulated using the Langmuir and Freundlich isotherm models. Their equations are shown in Equations (5) and (6):

(5) $$\mathrm{Langmuir}:\;q_{e}=\frac{q_{m}K_{L}C_{e}}{1+K_{L}C_{e}},$$

where *q_e_* (mg.g^−1^) is the adsorption capacity at equilibrium; *q_m_* (mg.g^−1^) is the theoretical maximum adsorption capacity; *C_e_* (mg.L^−1^) is the equilibrium concentration of the adsorbate; *K_L_* (L.mg^−1^) is the Langmuir adsorption constant.

Freundlich: *q_e_ = K_F_ C_e_*^1*/n*^, (6)

where *K_F_* ((mg.kg^−1^)/(mg.L^−1^)^n^) is the Freundlich adsorption constant; 1*/n* represents the intensity of adsorption (unitless).

## 3. Results

### 3.1. Physicochemical Properties of RHB and CSBC

**Figure 2:**
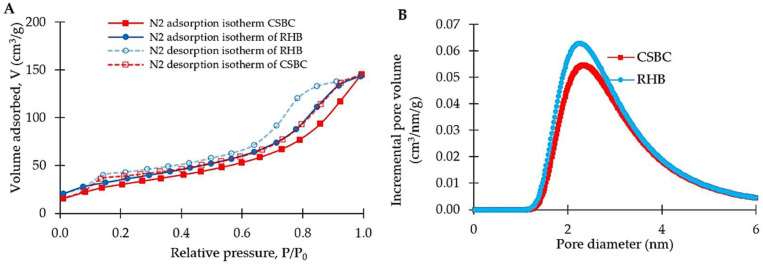
Nitrogen adsorption–desorption isotherms at 77 K (**A**) and pore size distribution (**B**) of RHB and CSBC.

The textural properties such as surface area, average pore size and pore volume were estimated from well-known calculation methods such as the Brunauer–Emmett–Teller (BET) and the Barrett– Joyner–Halenda (BJH) models. The nitrogen adsorption–desorption curves on RHB and CSBC are shown in Figure 2A, while the pore size distributions of RHB and CSBC estimated by the BJH method from desorption branches are shown in Figure 2B. In depth, at a low relative pressure P/P_0_ ≤ 0.1, the isotherms of RHB and CSBC exhibit the typical type I characteristic, where nitrogen adsorption increases linearly with the increase of relative pressure, indicating the existence of micropores. At medium relative pressures 0.5 < P/P_0_ < 0.9, an approximately closed hysteresis loop is formed and the isotherms of RHB and CSBC exhibit the type IV characteristic, demonstrating the existence of mesopores [28]. The desorption curves of RHB and CSBC are almost consistent with their adsorption curves, with differences existing at mid to high P/P_0_. According to the classification by the International Union of Pure and Applied Chemistry (IUPAC), the nitrogen adsorption–desorption isotherms of RHB and CSBC can be classified as being of type IV shapes and type H4 hysteresis loops, which indicates the existence of slit-shaped pores, attributed to mesoporosity [47]. This result can be further confirmed by the corresponding pore size distribution, as displayed in Figure 2B. The prepared RHB and CSBC possessed a mesoporous surface with a mean pore radius of approximately 2.26 nm and 2.34 nm, respectively.

It is obvious that the RHB had a higher adsorbed amount of nitrogen than the CSBC, implying that the RHB possesses a higher specific surface area and pore volume than CSBC. As a matter of fact, the specific surface area calculated using the BET equation was 115.59 m^2^.g^−1^ for RHB, slightly higher than that of CSBC with 107.97 m^2^.g^−1^. Chitosan-modified biochar produced from other feedstocks also exhibit lower surface areas than their unmodified biochar [37,43,45]. This is probably the reason for the blockage of partial pores by the incorporation of chitosan in the biochar matrix or the changes in the chemical compositions of the biochar surface so that nitrogen has less affinity for surface adsorption [37].

**Figure 3:**
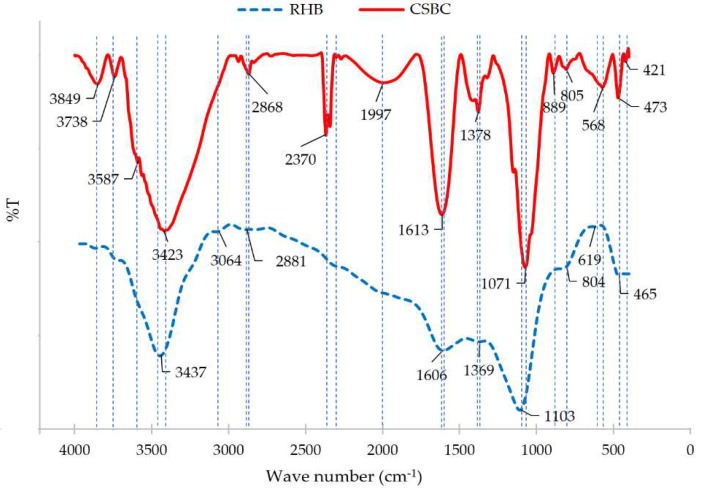
The FTIR analysis of RHB and CSBC.

The functional groups on the RHB and CSBC were detected through FT-IR analysis. Figure 3 show that the FT-IR spectra of CSBC were somewhat different from those of RHB. In particular, the FT-IR spectra of RHB in this study were similar to other high-temperature biochar derived from rice husk [48,49], which possess several types of functional groups, including O–H (3437 cm^−1^),—COOH (1606 cm^−1^), C–H aliphatic (1369 cm^−1^), C—O—C (1103 cm^−1^) and Si-O (465 cm^−1^) groups [50]. Meanwhile, characteristic bands for CSBC were observed in the infrared spectrum around 3849 cm^−1^ and 3423 cm^−1^, attributed to amine N–H symmetrical vibration and an H-bonded O–H group. In addition to these functional groups, the FT-IR spectrum of CSBC showed other typical infrared spectrums for chitosan, for example, sp^3^-hybridized C–H stretching vibrations of carbohydrate ring appeared at peak 2868 cm^−1^ [51], the amide II band (the N–H stretching vibrations) appeared at peak 1613 cm^−1^ [43] and the amide III band (the C–N or N–H stretching vibrations) appeared at peak 1378 cm^−1^ [52,53].

**Figure 4:**
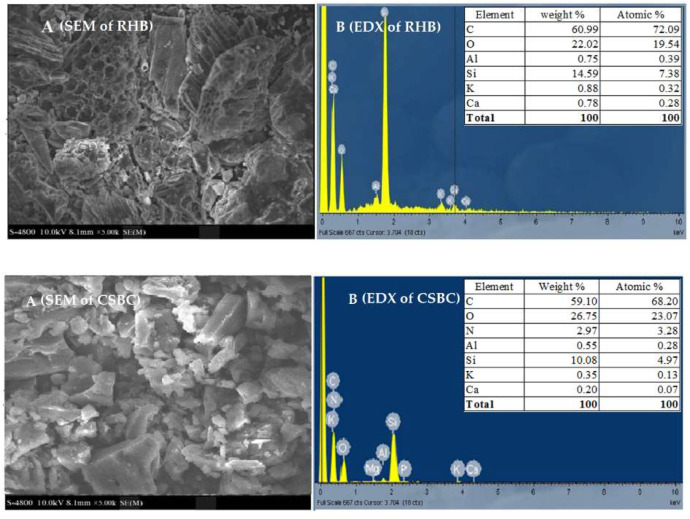
SEM-EDX analysis of RHB and CSBC; (**Figure A upper**): SEM of RHB; (**Figure B upper**): EDX of RHB; (**Figure A lower**): SEM of CSBC; (**Figure B lower**): EDX of CSBC.

The SEM morphological structures of CSBC and RHB are shown in Figure 4A. It is obvious that the surface morphology of precursor biochar significantly changed after modification with chitosan. The external morphology of CSBC generally possesses a highly irregular surface compared to the well-defined cylindrical pores of the RHB surface. These results were similar to other observations [40,54].

In the EDX results, both CSBC and RHB mainly consisted of C (59–61%), O (22–27%) and Si (10–15%) (Figure 4B). Significantly, the introduction of –NH_2_ groups into the chitosan-modified biochar hydrogel beads was confirmed by the appearance of a Nitrogen (N) peak (accounted for 2.97% atomic weight) after chitosan coating. The tapioca peel biochar modified with chitosan also reported similar findings [45].

From the FTIR and SEM-EDX results, it can be safely concluded that chitosan was successfully bonded with the biochar matrix. Based on the physical properties of CSBC and RHB, a feasibility test for MO removal was conducted to compare the performance between CSBC and RHB. The results are described in the following section.

### 3.2. pH-Dependent Mechanism

**Figure 5:**
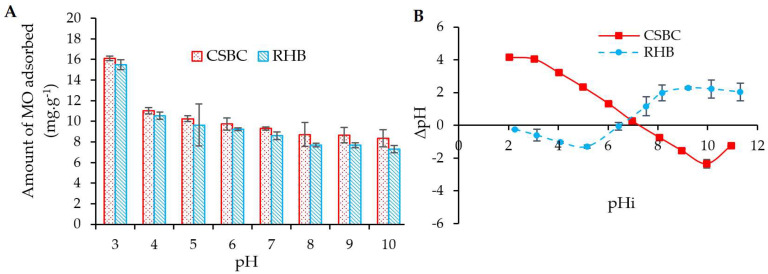
Effect of pH on MO adsorption (**A**) and pH_pzc_ of CSBC and RHB (**B**).

The effect of the solution pH on the adsorption capacity of MO by CSBC and RHB was performed in the range of 3–10 at room temperature (298 K), adsorbate dosage of 0.2 g, contact time of 240 min and initial CSBC/RHB concentration of 50 mg.L^−1^. Figure 5A show that MO adsorption onto CSBC and RHB was highly pH-dependent, where the amount of MO adsorbed into CSBC and RHB decreased with increasing solution pH. More specifically, as the initial pH increased from 3 to 10, the amount of MO adsorbed substantially decreased from 16.13 mg.g^−1^ to 8.35 mg.g^−1^, from 15.50 mg.g^−1^ to 7.30 mg.g^−1^ for CSBC and RHB, respectively.

The pH effect could be explained using the point of zero charge (pH_pzc_), the pH at which the surface of an adsorbent carries an equal amount of positive and negative charge. In comparison, the point of zero charge of CSBC (7.26) was slightly higher than the point of zero charge of RHB (6.48), as depicted in Figure 5B. The increase in the point of zero charge of CSBC is probably due to the addition of the basic groups (i.e., –NH_2_ and –OH groups) after chitosan was incorporated into the biochar matrix [43]. This finding was similar to other chitosan-modified biochar [40].

It is well recognized that when pH < pH_pzc_ (i.e., high H^+^ activity in solution), the RHB surface charges are more positively charged due to the protonation of the hydroxyl –OH group. In this case, a stronger electrostatic attraction between hydrogen (H^+^) with MO^–^ could occur; consequently, the amount of MO absorbed by RHB in an acidic environment was increased. For the CSBC case, in addition to the protonation of hydroxyl –OH groups at pH < pH_pzc_, the amine –NH_2_ group in the chitosan was also able to accept a proton from a hydronium ion (–NH_2_ + H_3_O + ⇆ NH_3_^+^ + H_2_O) [37]. As a result, electrostatic attraction between NH_3_^+^ and MO^−^ can easily occur; thus, CSBC generally shows better adsorption capacity for MO compared to RHB under the same adsorption conditions (Figure 5A).

Furthermore, the pK_a_ of MO was 3.46, and the MO molecule existed as a negatively charged species until pH 3.46. Afterwards, MO existed as neutral, and in high pH conditions, as slightly positive charged species. When pH > 4, the MO adsorption decreased with the increase of pH, which was partially caused by the competition between OH^−^ excess in the solution and anionic ions of MO. In brief, the pH-dependency of the employed materials can be explained by the electrostatic attraction between the negatively charged MO^−^ (solution pH > pK_a_) and the negatively charged surface of adsorbents (solution pH > pH_pzc_).

### 3.3. Adsorption Isotherms

The obtained optimum results used for kinetic and isotherm studies are shown in Figures S1–S3 (Supplementary Material). The optimum conditions for MO adsorption by both adsorbents were determined to be pH~3, a MO concentration of 50 mg/L, an adsorbent dosage of 0.2 g, adsorption time of 240 min and conditions at room temperature (298 K).

**Figure 6:**
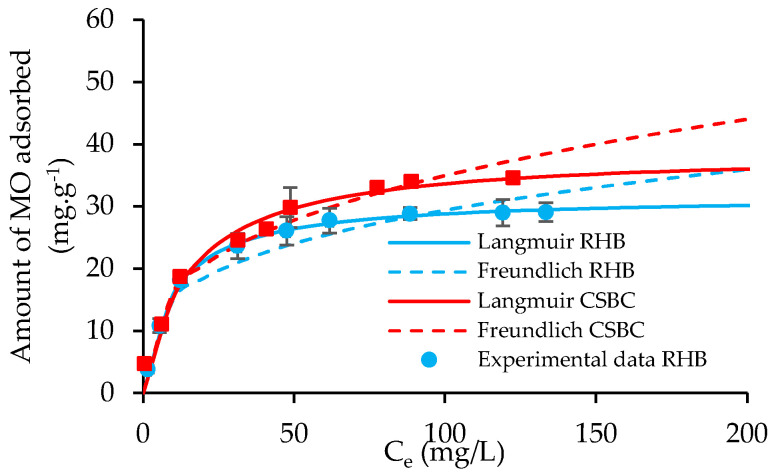
Fitting of Langmuir and Freundlich isotherms for MO adsorption on RHB and CSBC.

The nonlinear fitting curves of the Langmuir and Freundlich models are presented in Figure 6, while their isotherm parameters obtained from the adsorption experiments are tabulated in Table 1.

It is obvious that the experimental data of CSBC and RHB for MO adsorption has a trend to stimulate both Langmuir and Freundlich isotherm models, with the determination coefficient R^2^ of these models ranging from 0.92 to 0.99. However, a more in-depth comparison would reveal that the Langmuir model could fit the MO experimental data better, with R^2^ values close to 1 (0.998 for RHB and 0.980 for CSBC). The fitting of Langmuir suggested homogeneous adsorption for MO on the surface of RHB and CSBC. In addition, the relatively higher Langmuir affinity constant *K_L_* value of CSBC (*K_L_* = 0.17 L.mg^−1^) than that of RHB (*K_L_* = 0.10 L.mg^−1^) could be because of the stronger affinity of CSBC toward MO in aqueous solution.

Table 1 also report the maximum adsorption capacity of CSBC and RHB adsorbents obtained from the Langmuir model under the optimum adsorption conditions at room temperature (25 ± 2 °C), pH~3, time 240 min and adsorbent dosage of 0.2 g. It is recorded that the maximum adsorption capacity for MO of CSBC was estimated as 38.75 mg.g^−1^, comparatively higher than that of RHB with 31.63 mg.g^−1^.

### 3.4. Adsorption Kinetics

**Figure 7:**
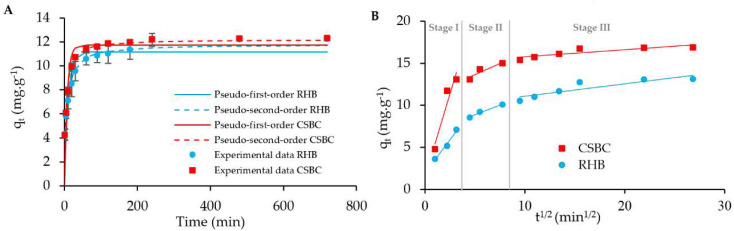
Fitting of PFO and PSO kinetic (**A**) and IPD (**B**) models for MO adsorption on CSBC and RHB.

Three well-known theoretical kinetic models, including pseudo-first-order (PFO), pseudo-second-order (PSO) and intraparticle diffusion (IPD), were employed, and their derived coefficient of determination R^2^ was used to obtain the best-fitting kinetic model for the experimental data. The experimental data (*q_t_*, mg.g^−1^) of RHB and CSBC toward MO fitted with the PFO and PSO kinetic models are displayed in Figure 7A, while the fitting for the IPD kinetic model is depicted in Figure 7B. The calculated kinetic parameters for MO adsorption onto RHB and CSBC are summarized in Table 2.

According to Table 2, the PSO kinetic model was better fitted to the experimental data than the PFO model for both tested materials, where the R^2^ values obtained by the PSO kinetic model were greater than the R^2^ values from the PFO kinetic. In depth, the R^2^ value of the PFO kinetic equation was 0.86 for CSBC and 0.83 for RHB, while that of the PSO kinetic model was 0.94 for CSBC and 0.93 for RHB. In addition, the adsorption capacity values (q_e,cal_) calculated by PSO kinetics (11.81 mg.g^−1^ for RHB, 12.02 mg.g^−1^ for CSBC) showed closer values to those obtained by experiments (q_e,exp_) than PSO kinetics (12.16 mg.g^−1^ for RHB, 12.20 mg.g^−1^ for CSBC), revealing that the obtained data is well-fitted by the PSO kinetic model. The PSO model comes as an indication that chemisorption might be the rate-limiting step in the adsorption process of methyl orange. A good fit to the PSO kinetic model was also observed in the other study on malachite green and Rhodamine B adsorption by chitosan–tapioca peel biochar [45].

The experimental kinetic data were further analyzed with the IPD model, in which a linear plot of *q_t_* vs. t^1/2^ was used to obtain K_d_ and C constants. Figure 7B demonstrate that the adsorption process of MO by RHB and CSBC was not a straight line throughout the whole experiment duration, involving three different kinetic stages: the first external film diffusion stage (stage I), the second gradual adsorption stage (stage II) and the third equilibrium adsorption stage (stage III). The R^2^ values obtained from the three stages were found between 0.86 and 0.97, indicating that the IPD mechanism could play an important role in the MO adsorption process but not the sole rate-limiting step (C_1_, C_2_, C_3_ ≠ 0) [29]. In fact, the obtained diffusion rates k_id1_ > k_id2_ > k_id3_ indicated that external film diffusion could play an important role in the rate-limiting step during diffusion [55]. A comparison of the external diffusion rate constants k_id1_ between the CSBC (3.94) and RHB (1.58) in Table 2 demonstrate that faster external diffusion occurred in CSBC than in RHB, which could be attributed to the higher swelling behavior of chitosan hydrogel beads [55].

### 3.5. Mechanism Exploration and Comparison Assessment

In light of the above kinetic analysis, it is apparent that the adsorption process of MO by RHB and CSBC hydrogel beads followed both PSO and IPD kinetic models, advising that both chemisorption and diffusion mechanisms were the rate-controlling steps over the whole adsorption process.

**Figure 8:**
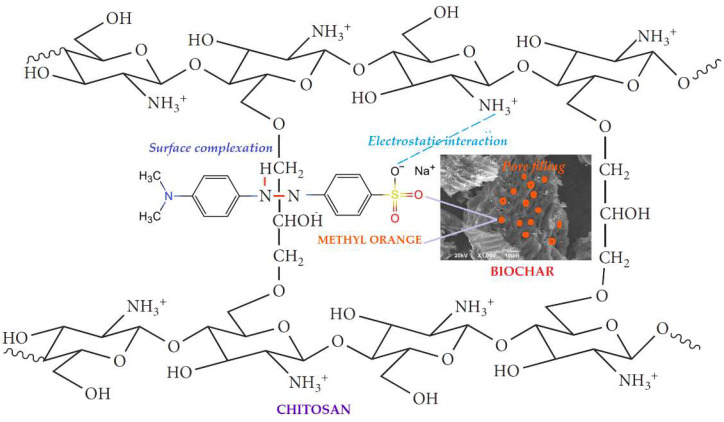
Possible chemisorption and diffusion mechanisms of MO onto CSBC hydrogel beads.

In the chemisorption mechanism of RHB, the adsorption of MO was carried out mainly by the electrostatic interactions between the positively charged surface (i.e., H^+^) of RHB and the negatively charged (i.e., SO_3_^−^) of MO. In the chemisorption mechanism of CSBC, the adsorption mechanism predicted would be the contribution of individual material (biochar and chitosan) in chitosan-modified biochar combinations. As demonstrated previously, the modification process by chitosan successfully incorporated the –NH_2_ and –OH groups into the biochar matrix. The incorporation of these new functional groups is helpful in stimulating the formation of complexes between the MO molecules and CSBC material (illustrated in Figure 8). Thus, in addition to the electrostatic interaction mechanism, the surface complexation mechanism may account for the contribution of chitosan to the CSBC, thereby enhancing the adsorption efficiency of the CSBC material to 1.3 times as compared to RHB (38.75 mg.g^−1^ for CSBC and 31.63 mg.g^−1^ for RHB).

Thus, surface complexation and electrostatic interaction mechanisms may account for the chemisorption of MO ions by CSBC, thereby enhancing the adsorption efficiency of the CSBC material. As found in the present study, the adsorption capacity for MO of CSBC (38.75 mg.g^−1^) was close to 1.3 times greater than BC (31.63 mg.g^−1^). Several studies that were conducted on chitosan-modified different raw materials of biochar, such as peels of pomelo [56], apple branches [39], pine wood [43] and corncob [57], have shown that the use of chitosan-modified biochar also contributed to the overall adsorption performance, as listed in Table 3. Although the adsorption capacity of CSBC does not seem to be significantly improved regarding RHB as compared with other studies (Table 3), it is true that no safe comparisons can be made here because the feedstock used, the pyrolytic temperature, the experimental conditions and target substrates are totally different.

## 4. Conclusions

Chitosan-modified biochar hydrogel beads were successfully synthesized in this study. The batch experimental study demonstrated that chitosan beads coated onto biochar matrix could enhance the adsorption capacities of methyl orange molecules from the aqueous solution since chitosan modification helps increase the number of functional groups (i.e., −NH_2_ and −OH groups). The theoretical maximum adsorption capacities of the chitosan-modified biochar were close to 1.3 times greater than unmodified biochar (38.75 mg.g^−1^ for CSBC and 31.63 mg.g^−1^ for RHB), which were obtained at room temperature (25 ± 2 °C), pH~3, time 240 min and adsorbent dosage 0.2 g. The experimental data were well described by the Langmuir isotherm, with R^2^ values close to 1 (0.998 for RHB and 0.980 for CSBC), suggesting homogeneous adsorption for MO on the surface of RHB and CSBC. The kinetic data for MO adsorption was well-fitted to both pseudo-second-order and intra-particle diffusion kinetic models, indicating that the chemisorption mechanism and intra-particle diffusion mechanism may govern the adsorption process of methyl orange molecules by both RHB and CSBC adsorbents. Briefly, the developed chitosan-modified biochar hydrogel beads can be utilized as an effective adsorbent for the removal of methyl orange molecules from aqueous solution.

## Figures and Tables

**Table 1:** Fitting results of Langmuir and Freundlich isotherms for MO adsorption by RHB and CSBC.

Model	Isotherm Parameter	Adsorbent	
		RHB	CSBC
*Langmuir*	*q_m_* (mg.g^−1^)	31.63	38.75
	*K_L_* (L.mg^−1^)	0.10	0.17
	R^2^	0.998	0.980
*Freundlich*	1/n	3.45	3.02
	*K_F_* ((mg.kg^−1^)/(mg.L^−1^)^n^)	7.73	7.89
	R^2^	0.916	0.974

**Table 2:** Fitting results of different kinetics for MO Adsorption by RHB and CSBC.

Adsorbent	q_e,exp_ (mg.g^−1^)	Kinetic Model							
		*Pseudo-First-Order*				*Pseudo-Second-Order*			
		q_e,cal_ (mg.g^−1^)		k_1_ (1.min^−1^)	R^2^	q_e,cal_ (mg.g^−1^)	k_2_ (g.mg^−1^.min^−1^)	R^2^	
**CSBC**	12.20	11.72		0.13	0.864	12.02	0.02	0.942	
**RHB**	12.16	11.16		0.10	0.832	11.81	0.01	0.930	
	*Intra-particle diffusion*								
	Stage I: t = 1–10 min			Stage II: t = 20–60 min			Stage III: t > 60 min		
	C_1_	k_id1_	R^2^	C_2_	k_id2_	R^2^	C_3_	k_id3_	R^2^
	mg.g^−1^	mg.g^−1^.min^−1/2^		mg.g^−1^	mg.g^−1^.min^−1/2^		mg.g^−1^	mg.g^−1^.min^−1/2^	
**CSBC**	1.47	3.94	0.927	10.95	0.54	0.926	14.94	0.08	0.858
**RHB**	1.92	1.58	0.970	6.59	0.46	0.974	9.63	0.14	0.891

**Table 3:** Effect on removal capacity by chitosan coated on biochar matrix.

Feedstock	Pyrolytic Temperature (°C)	Surface Area (m^2^ g^−1^)	Experimental Conditions	Contaminants	Effect on Removal Capacity	Ref.
Peels of pomelo	450	Not available	pH~3; T = 298 K, C_0_ = 10 ÷ 50 (mg/L)	Ciprofloxacin	Near 11 times higher than unmodified biochar (q_max,CSBC_ = 36.72 mg.g^−1^)	[56]
Apple branches	300	S_BC_ = 13.3; S_CSBC_ = 23.3	pH~9; T = 298 K, C_0_ = 10 ÷ 500 (mg/L)	Phloridzin	Near 2 times higher than unmodified biochar (q_max,CSBC_ = 28.96 mg.g^−1^)	[39]
Pinewood	425	S_BC_ = 10.5; S_CSBC_ = 7.13	pH~5; T = 318 K, C_0_ = 50 ÷ 350 (mg/L)	Pb(II)	Near 3 times higher than unmodified biochar (q_max,CSBC_ = 134 mg.g^−1^)	[43]
Corncob	400	S_BC_ = 301.9; S_CSBC_ = 631.5	pH~12; T = 298 K, C_0_ = 10 ÷ 100 (mg/L)	Methylene Blue	Near 2 times higher than unmodified biochar (q_max,CSBC_ = 499.8 mg.g^−1^)	[57]
Rice husk	500	S_BC_ = 115.59; S_CSBC_ = 107.97	pH~3; T = 298 K, C_0_ = 10 ÷ 200 (mg/L)	Methyl Orange	Near 1.3 times higher than unmodified biochar (q_max,CSBC_ = 38.75 mg.g^−1^)	This study

## Data Availability

Not applicable.

## Supplementary Materials

The following supporting information can be downloaded at: https://www.mdpi.com/article/10.3390/toxics10090500/s1, Figure S1: The effects of RHB and CSBC dosage on MO adsorption; Figure S2: The effects of initial MO concentration on adsorption by RHB and CSBC; Figure S3: The effects of contact time on MO adsorption onto RHB and CSBC.
