# Supplementary material for: Chitosan-Modified Biochar and Unmodified Biochar for Methyl Orange: Adsorption Characteristics and Mechanism Exploration
Source: Toxics. 2022 Aug 27;10(9):500. doi: 10.3390/toxics10090500 (PMC9501881; doi:10.3390/toxics10090500)
Supplement: Supplementary file 1 [file toxics-10-00500-s001.zip › toxics-1872125-supplementary.pdf]

toxics

MDPI

Article

Chitosan-Modified Biochar and Unmodified Biochar for Methyl Orange: Adsorption Characteristics and Mechanism Exploration

Nguyen Xuan Loc [1,†], Phan Thi Thanh Tuyen [1], Le Chi Mai [2] and Do Thi My Phuong [2,*,†]

[1] Department of Environmental Sciences, College of the Environment and Natural Resources, Can Tho University, Can Tho 900000, Vietnam
[2] Department of Environmental Engineering, College of the Environment and Natural Resources, Can Tho University, Can Tho 900000, Vietnam
* Correspondence: dtmphuong@ctu.edu.vn; Tel.: +84-91-918-8834
† These authors contributed equally to this work.

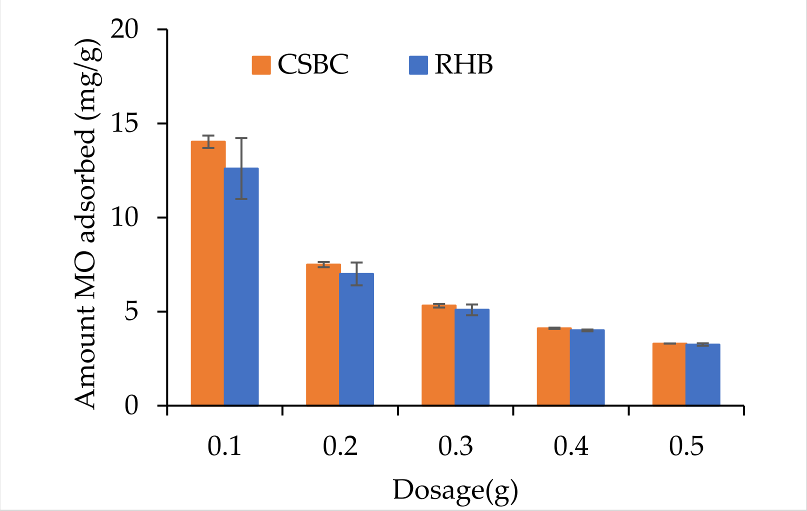

Figure S1. The effects of RHB and CSBC dosage on MO adsorption.

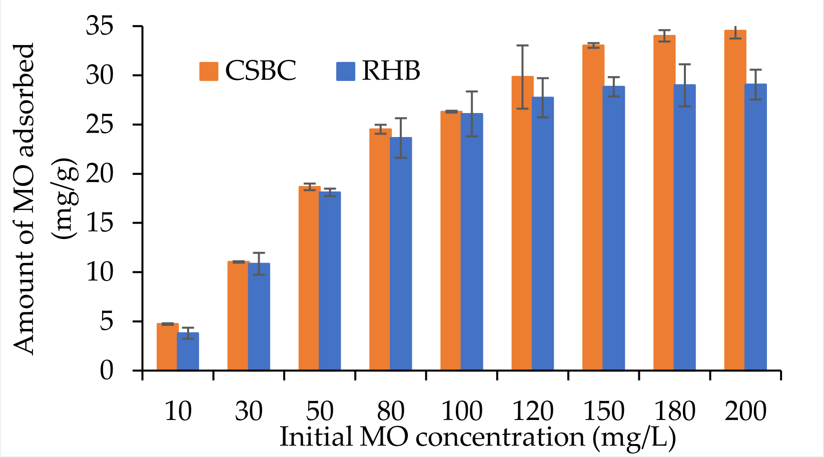

Figure S2. The effects of initial MO concentration on adsorption by RHB and CSBC.

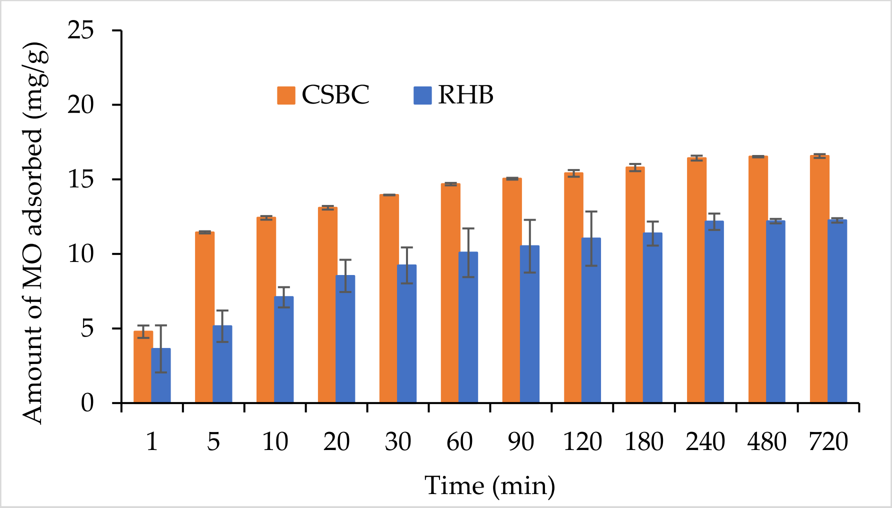

Figure S3. The effects of contact time on MO adsorption onto RHB and CSBC.
